# Influence of Unperceived Victimization on Adolescent Well-Being

**DOI:** 10.3390/children11101183

**Published:** 2024-09-27

**Authors:** Elisa Larrañaga, Maria Carmen Cabrera, Santiago Yubero

**Affiliations:** Department of Psychology, University of Castilla-La Mancha, 1601 Cuenca, Spain; carmen.cherrera@uclm.es (M.C.C.); santiago.yubero@uclm.es (S.Y.)

**Keywords:** bullying, victims, unperceived victimization, psychological distress, students, adolescence

## Abstract

**Background:** Bullying has been shown to have negative consequences on the health and well-being of adolescents. Some students may experience various bullying behaviors and not consider themselves victims, finding themselves in a situation of unperceived mistreatment. Few studies have focused on the relationship between self-reported victimization behaviors and self-identification as a victim. Our objective is to determine the prevalence of unperceived victimization and analyze its relationship with adolescent distress. **Methods:** A total of 964 adolescents aged between 12 and 19 years participated. Standardized instruments were used to assess psychological distress, bullying in the last month and previous years, perception of victimization, and resilience. **Results:** More than 20% of adolescents are in a situation of unperceived victimization, not recognizing themselves as victims despite reporting bullying behaviors. Among girls and middle adolescence, perceiving oneself as a victim increases the likelihood of feeling distress. The likelihood of experiencing distress increases with previous victimization and decreases with high resilience. **Conclusions:** Mental health professionals should consider bullying as a factor of distress in adolescence. It is essential to refer adolescent victims of bullying to specialized mental health resources to adequately address their well-being and increase resilience as a protective factor.

## 1. Introduction

The World Health Organization highlighted the need to address adolescent health, as this is a particularly vulnerable period for mental health [1]. The estimated prevalence of mental health problems among adolescents is 13.4% [2]. In Spain, a recent study estimated a 7.7% risk of subclinical psychological problems among adolescents [3].

To effectively address adolescent health needs, it is necessary to analyze the determinants that influence well-being to achieve Sustainable Development Goal 3 set by the United Nations General Assembly in 2015: ensure healthy lives and promote well-being for all at all ages [4].

Satisfactory social relationships promote positive adolescent development. Therefore, the school climate is a significant predictor of students’ psychological adjustment [5]. Indeed, the school climate can foster positive development and life satisfaction, but it is also a context where various forms of conflict, such as bullying, can occur. Bullying is intentional and repetitive aggressive behavior against someone who cannot defend themselves. It can involve physical, verbal, or social exclusion attacks; when technology is used to bully, it is called cyberbullying [6].

### 1.1. Unperceived Victimization

To measure bullying behaviors, self-report measures can be used, where various bullying behaviors are presented, and each person responds based on their experience. Other studies have used peer nomination measures, where individuals report on their peers’ involvement by identifying victims and aggressors. Correlations between these measures are usually low to moderate, with discrepancies between self-report and peer nominations [7,8,9].

Connell et al. examined the correspondence between reported bullying experiences and the perception of being a victim, finding that 34% of the sample reported a lack of correspondence between experiences and perceptions [10]. Some students report their victimization experiences but do not perceive themselves as victims, a situation known as unperceived victimization. A study with Spanish primary school students found a 36% rate of self-reported victimization compared to a 24% perception of being a victim, with no significant differences by gender [11]. No studies have analyzed unperceived victimization in secondary education.

Consequently, the first objective of this study was to explore unperceived victimization among adolescents.

### 1.2. Relation between Victimization and Subjective Well-Being

Bullying is a problem for minors with severe emotional consequences [12], affecting health and the educational environment [13,14]. Evidence shows a reduction in psychological well-being among bullying victims. Specifically, they may exhibit symptoms of depression [15], anxiety, stress [16], lower social support, substance use [17], and poor academic performance [18]; leading to a deterioration in adolescent well-being [15]. Additionally, the effects of victimization on well-being persist over time, even years after the bullying occurs [19], potentially extending into university [20,21] and adulthood [22].

These results imply that it is necessary to analyze bullying behaviors in the present and past due to their short- and long-term consequences on well-being. The discrepancy in the perception of bullying experiences (defined as the discrepancy between self-reported bullying experiences and those reported by peers) has shown negative consequences on adolescent well-being [8]. These results indicate that not only those who experienced bullying but also those whose perceptions differed from their peers (those who reported experiencing bullying, although not supported by peer reports) also showed lower well-being.

Research in gender-based violence, which is the field where the situation of unperceived violence has been most analyzed, has shown that it is unlikely that victims will seek help if they do not consider themselves to be experiencing a situation of violence [23]. However, it is also relevant due to its implication in the commitment to intervention and intention to change [24,25,26]. Furthermore, the lack of victim self-awareness can lead, in the long term, to the perpetuation of distress and, consequently, the maintenance of victimization [27].

No studies have reported on the relationship between unperceived bully victimization and well-being. The second objective of this study was to examine the relationship between feeling or not feeling like a victim and distress, considering the information provided about the bullying received.

### 1.3. Resilience, Bullying, and Subjective Well-Being

Not all victims are equally affected [28]. Systematic reviews have highlighted the relevance of individual protective factors [29,30]. Resilience has stood out among the factors that facilitate adolescents’ adaptation despite experiencing bullying [31,32,33]. Resilience is defined as a personality trait that buffers the negative impact of stressful events, promoting the individual’s adaptation to the situation [24]. Empirical evidence has revealed that resilience could buffer the negative influences of bullying victimization on well-being. Research results link low resilience levels with greater involvement in bullying, both as victims and aggressors [34,35]. Hinduja and Patchin noted that youth with higher resilience levels were not only less bullied by their peers but also experienced fewer consequences of bullying [31]. More resilient bullying victims could recover from bullying victimization and achieve positive adaptation quickly because they can use more and more effective resources to cope with bullying victimization [36,37]. Consequently, their well-being may be less affected by the harmful effects of bullying victimization. Resilience has also shown associations between victimization and well-being in polyvictimization [38] and chronic victimization [21]. It is also a protective factor for adolescent bullying victims against suicide [16]. Thus, resilience can offset the negative effects of bullying victimization on well-being [39].

Undoubtedly, resilience is closely related to adolescents’ mental health, and it is necessary to give this variable a prominent role in prevention and clinical care programs [40], including bullying cases [41].

The third objective of this study was to analyze the role of resilience in the relationship between the perception of victimization and the distress of victims.

### 1.4. Current Study 

Few studies have focused on the perception of victimization, on the relationship between self-reported victimization behaviors and the self-ascription of being a victim. This is important for intervention because the perception of being a victim plays a significant role in recognizing the experience of bullying and, therefore, in seeking help [28]. The authors also suggest that further research is needed on the role played by resilience in the face of victimization behaviors [42]. Our objectives were: 1—to analyze the prevalence of unperceived victimization in adolescents, 2—to study the influence of the perception of victimization on adolescent distress, 3—to examine the association of the perception of victimization with psychological distress, taking into account bullying variables and the role of resilience as a protective factor. Based on data obtained in primary education [11], our study hypothesis has been that in adolescents there will appear a group of students who are in a situation of unperceived victimization, who do not conceptualize the aggressive behaviors received as bullying. We did not propose prior hypotheses for the second and third objectives, as there are no previous studies that could indicate a direction of the relationship. Therefore, this part will be exploratory.

## 2. Materials and Methods

### 2.1. Participants and Procedure

This cross-sectional study was conducted in 6 public school classrooms in Castilla-La Mancha. The sample was incidental, consisting of 964 adolescents aged 12–19 years, with 447 boys and 517 girls, who completed all items of the research questionnaires. Regarding age, according to the stages of adolescence (early adolescence—between 10 and 13 years old, middle adolescence—from 14 to 17 years old, and late adolescence—over 17 years old) [43], 277 are in early adolescence, 636 are in middle adolescence, and 80 are over 17 years old.

The study was conducted in accordance with the Declaration of Helsinki. Data were collected after obtaining informed and written consent from all parents of the participating students and the students themselves. During a 45-min class session and in the presence of a researcher, students were informed about the study’s objectives and asked to voluntarily and anonymously complete the various questionnaires, with the option to withdraw from the study at any time.

### 2.2. Measures

Psychological Distress Scale (K10) [44]. This scale was validated in the Spanish population [45]. The total score can indicate the risk of psychological distress in the past month. It consists of 10 items with a five-point Likert response format. Using this scale, participants are asked to indicate the extent to which they agree with the 10 statements presented to them (for example, “you feel depressed?”, “you feel nervous?”). Each item is scored using the following response options: 1 (never), 2 (rarely), 3 (sometimes), 4 (many times), and 5 (always). The scale ranges from 10 to 50, with higher scores indicating greater distress. The scale’s reliability was *α* = 0.87.

Self-report. Bullyharm [46]. This scale was validated in the Spanish population [47]. Comprising 14 Likert-type items on bullying behaviors with four response options from 0 (it has not happened to me) to 3 (it has happened 2 or more times a week). An example item is, “Called me a bad name” or “Excluded me from their group”. Responses are based on behavior in the past month. Internal consistency was adequate: physical *α* = 0.76, verbal *α* = 0.77, exclusion *α* = 0.76, and cyberbullying *α* = 0.79. The self-reported bullying score was dichotomized following the same criteria as in previous research [48]. Participants scoring above one on at least one victimization item were classified as victims of bullying.

Previous Victimization: Measured using the Yubero et al. questionnaire [49], asking about victimization in previous years. An example item is, “I have been insulted, called names, etc.” Reliability was α = 0.74.

Self-nomination: Included a self-report measure of victimization through a direct question to determine if they perceived themselves as victims of bullying. Students responded with two alternatives, yes or no [10,11]. Previous research considering both self-report and peer nomination measures defined the discrepancy in bullying experience perception by the concordance or discordance between them. In our case, the discrepancy also comes from the concordance or discordance of information provided between self-reported bullying behaviors and self-nomination as a victim. We have identified four groups. Consistent Victims: Adolescents with consistent information in both bullying measures, reporting victimization behaviors, and perceiving themselves as victims. Unperceived Victims: Adolescents who report victimization behaviors but do not perceive themselves as victims. Inconsistent Victims: Adolescents who do not report victimization behaviors but perceive themselves as victims. Non-Victims: Adolescents who are not victims, do not report victimization behaviors, and do not perceive themselves as victims.

Resilience: Assessed with the abbreviated CD-RISC scale, validated in its Spanish version adapted for adolescents [50]. It includes 10 items rated on a Likert scale measuring frequency from 0 (not true at all) to 4 (true nearly all the time). An example item is, ‘Coping with stress can strengthen me.’ The scale ranges from 0 to 40, with higher scores indicating a higher level of resilience. The scale’s reliability was *α* = 0.85.

### 2.3. Data Analysis

First, contingency analyses examined the prevalence of nonperceived victimization and its distribution by gender. Additionally, the frequency distribution in the study groups is presented in the measures section. Secondly, through analysis of variance, the relationship with distress was analyzed according to the perception of victimization, considering gender (4 × 2) and the stage of adolescence (4 × 3). Finally, the relationship between the study variables was examined through a Pearson correlation analysis, including the difference between the variables according to the segmentation variables: gender and age; and linear regression analyses segmented by gender and age were conducted to analyze the impact of the study variables on adolescent distress. Data processing was performed using the SPSS 29.0 statistical program.

## 3. Results

### 3.1. Prevalence of Unperceived Victimization

A total of 27.9% reported experiencing bullying behaviors at least once a week, with a higher total percentage of victims among boys (30.8% boys, 25.5% girls; *χ*^2^ = 3.43, *p* < 0.05), and in early adolescents (31.5% early adolescence, 28.8% middle adolescence, 11.4% late adolescence; *χ*^2^ = 12.58, *p* < 0.05). When asked about their perception of victimization, 7% responded affirmatively. Therefore, more than 20% of adolescents do not recognize themselves as victims despite reporting bullying behaviors from their peers. The percentage of students in a situation of unperceived victimization is higher among boys, 26% (*n* = 115), compared to girls, 20.3% (*n* = 106) (*χ*^2^ = 4.13, *p* < 0.05). It decreases significantly with age: early adolescence, 24.5% (*n* = 66); middle adolescence, 23.9% (*n* = 147); late adolescence, 10.1% (*n* = 8) (*χ*^2^ = 7.61, *p* < 0.05).

Table 1 shows the distribution in the four analysis groups according to gender and age. 

### 3.2. Perception of Victimization and Distress

The analysis of the simple effects of the perception of victimization (*F* = 29.13, *p* < 0.001, *η*^2^ = 0.084) shows that adolescents in a situation of unperceived victimization reported the same level of distress as consistent victims (*p* < 0.001).

The distress results (Table 2) show a significant effect of gender (*F* = 16.57, *p* < 0.001, *η*^2^ = 0.017) with higher distress among girls, and a significant increase in distress according to the perception of victimization (*F* = 31.83, *p* < 0.001, *η*^2^ = 0.091). There is no interaction between the two variables (*p* = 0.704).

The distress results (Table 3) show a significant effect of age (*F* = 3.75, *p* < 0.05, *η*^2^ = 0.010) with higher distress as age increases and a significant increase in distress according to the perception of victimization (*F* = 10.10, *p* < 0.001, *η*^2^ = 0.032). There is no interaction between the two variables (*p* = 0.810).

### 3.3. Relations between Study Variables

All the variables of victimization included in the study are significantly correlated with distress in a positive direction. Resilience is negatively related to distress. Girls have reported more exclusion bullying, while boys have reported more physical bullying. Boys report higher levels of resilience than girls. No significant differences are found in any variable according to age (Table 4).

### 3.4. Relationship between Perception of Victimization and Distress by Gender

In the regressions for both boys and girls, previous victimization appears as a risk factor for distress, while resilience acts as a protective factor. A differential element is the perception of victimization, which only reaches significance in the analysis for girls. The type of bullying related to distress also varies, for boys, verbal bullying is significant, while for girls, exclusion is significant (Table 5).

### 3.5. Relationship between Perception of Victimization and Distress by Age

In the regressions for age, previous victimization appears as a risk factor for distress in early and middle adolescence, while resilience acts as a protective factor for all adolescents. A differential element is the perception of victimization, which only reaches significance in the analysis for middle adolescence. The type of bullying related to distress is also significant in the exclusion of middle adolescents (Table 6).

## 4. Discussion

Bullying has shown negative consequences on the health and well-being of adolescents [15]. In Spain, 7.7% of adolescents are at risk of mental health difficulties [3]. The issue of bullying as a source of these difficulties cannot be overlooked.

In primary education students, a group has reported experiencing bullying behaviors but did not identify as victims [11]. This is termed unperceived maltreatment or unperceived victimization. No studies have analyzed unperceived victimization in adolescents or its role in subjective distress.

Like primary students, a group of adolescents reported bullying behaviors but did not identify as victims. The first objective was to study the prevalence of unperceived victimization in adolescents. Results show that 20% of adolescents who reported bullying behaviors do not see themselves as victims, with a higher percentage of boys in this category. We can consider that they do not interpret the behaviors they receive as bullying [51]. This result suggests that victimization behaviors may be normalized among adolescents. In previous studies on dating violence experiences, discrepancies also appear between the abuse reported by girls and what they perceive, as they only identify the most severe cases as abuse [52]. In late adolescence, unperceived victimization decreases. Previous research has found a reduction with age in children’s perception of having been victims [11]. This can be interpreted as a change in the concept of bullying, including repetition and intentionality [53], which could also affect self-admission of victimization [54]. As adolescence progresses, young people focus more on their peers [55], leading to greater openness to peer relationships in middle adolescence [56]; therefore, it could also be due to a greater concern for their image. If older adolescents consider that being a victim affects their social image, they would be less willing to consider themselves victims. Another reasoning could be the justification of violence, but no significant differences have been found in the justification of violence between the ages of 14 and 18 [57,58].

Discrepancies between self-reported bullying experiences and those reported by peers have shown negative consequences on adolescent well-being [8]. However, there is no prior research on how discrepancies between self-reported experiences and self-identification as a victim affect well-being. This study aims to explore the relationship between victimization perception and distress.

Consistent with previous research, nonvictimized adolescents reported significantly lower distress levels [59,60], and girls showed higher psychological distress than boys [61,62]. The results indicate a significant increase in distress among adolescents experiencing bullying behaviors, regardless of their self-perception as victims. In partner violence, nonperception of maltreatment is also associated with the same health problems as perception [63].

Adolescents who perceive themselves as victims but do not experience victimization at the time of the survey also reported higher distress than noninvolved adolescents. The distress caused by being a victim can persist over time [19].

This finding is reinforced by the regression results, which show that the likelihood of experiencing distress increases with previous victimization experiences. Previous research has confirmed the stability of victimization. For some minors, victimization at school may mark the beginning of a lifelong history of victimization [59,64].

For girls, being a victim of exclusion, and for boys, being a victim of verbal bullying, increases the likelihood of feeling distress. Research has shown the negative effects of exclusion on well-being [37,65,66]. There is evidence that girls place more importance on the school climate and peer relationships than boys [67]. Exclusion leads to social rejection and isolation [37], which can cause greater distress in girls. Verbal victimization can damage adolescents’ social standing and, consequently, their self-esteem [68]. Additionally, self-concept also influences the well-being of bullying victims [33]. In light of these results, health specialists should consider the bullying behaviors that adolescents have experienced and/or may be experiencing, by collecting a history of school bullying.

Self-nomination reached significance only in the equation for girls, increasing the likelihood of feeling distress with the self-nomination of being a victim. Previous research has highlighted that girls more frequently use maladaptive strategies to cope with bullying, such as rumination [69,70]. Rumination is a passive thinking style that involves continuously thinking about problems [71], which affects the ability to adopt positive solutions and increases distress [72,73]. The perception of being a victim may increase this behavior in girls, leading to increased distress.

A high capacity for resilience reduces the likelihood of distress. Resilience has become significant as a protective factor against distress in all stages of adolescence, for both boys and girls. Results that align with previous research have shown that resilience allows for positive adaptation to difficulties, such as bullying [31,32,33], mitigating distress, as well as other negative emotions caused by bullying and cyberbullying [39,74]. Additionally, it helps improve young people’s social relationships and develop properly, maturing into competent adults [75] despite adverse experiences [76]. These results highlight the importance of fostering resilience from early adolescence as a protective factor against bullying victimization.

These findings could contribute to improving prevention programs for the adolescent population. Strengthening resilience could prevent the consequences of distress [33,39]. Additionally, distress is a risk factor for revictimization [32,77] and for perpetrating bullying [15,16]. Therefore, interventions should consider reducing psychological distress through all educational, social, and health professionals.

At a practical level, these findings could contribute to the improvement and/or supplementation of current prevention and intervention programs for bullying among adolescents. The results of this research show implications for the design of interventions capable of protecting and promoting the psychological well-being of adolescents. Firstly, it is essential to address bullying within the framework of health network institutions, including pediatric teams [78]. The results of this work justify greater attention to the clinical care of adolescents reporting victimization behaviors, regardless of whether they identify as victims of bullying. Although the perception of being a victim increases distress, we have seen that adolescents who suffer victimization but do not perceive themselves as victims also show higher levels of distress. Not perceiving oneself as a victim may lead to not seeking help, thus maintaining or even increasing the distress. It is important to note that distress itself is a risk factor for revictimization, placing them in a very problematic future position. Early intervention seems relevant to avoid later repercussions on well-being. This intervention needs to reinforce resilience to cope with problematic situations and enhance positive strategies for dealing with bullying, seeking social support, and developing socioemotional skills. In this regard, the role of teachers is fundamental in helping them recognize bullying situations and providing effective strategies [79]. However, we must call for the intervention of health professionals who can adequately assess the distress of adolescents and consider victimization behaviors as a potential source of conflict for their positive well-being development. As we have seen, previous experiences of bullying victimization and present experiences influence adolescents’ distress. We cannot restrict health intervention only to adolescents who perceive themselves as victims; it is necessary to provide support and healthcare to all minors involved in bullying victimization behaviors, regardless of their recognition as victims.

Developing adolescents’ resilience can be a positive way to mitigate the effects of bullying on distress. Therefore, it would be important to include actions to reinforce resilience capacity in prevention programs carried out in educational centers. Additionally, it should be a fundamental pillar in intervention programs with bullying victims.

Furthermore, it is necessary to work with adolescents on recognizing bullying in all its forms and its consequences on their health because adolescents may not perceive the connection between their behaviors and the consequences [80]. There are previous experiences of working through reading to prevent bullying that address these aspects and would be of interest to consider at this stage of development [81].

Adolescents continue to suffer from bullying, which is affecting their psychological well-being [67,82,83]. Well-being in the present time and possibly in their future well-being. It is urgent to adopt clinical measures that can help adolescents have positive development.

For future lines of research, it is relevant to study the influence of gender on the perception of victimization, both for its role in recognizing different forms of bullying and in coping strategies for victims’ distress, to achieve greater intervention effectiveness. It would also be of interest to consider polyvictimization, as it has been shown to be a risk factor for well-being [38]. Additionally, it would be interesting to jointly analyze the influence of other school-related consequences of bullying, such as absenteeism and low performance, as they also impact adolescents’ well-being. Bullying research uses control theory to understand adolescents’ involvement in bullying behaviors [84]. Understanding the use of resource control strategies can help clarify the phenomenon of unperceived victimization.

### Limitations

Firstly, the results obtained are not generalizable to students of other educational levels. We have not considered the identification of adolescents with disabilities; it would be important to take this into account in future research on the perception of victimization. Secondly, this research only used self-report measures, which may introduce bias due to social desirability. Additionally, the instrument used to measure bullying is a subscale containing a limited number of items, and the participation percentages depend on the behaviors recorded. Therefore, it would be interesting to replicate the research using scales that capture a broader range of behaviors. Furthermore, the cross-sectional nature of this study prevents causal inferences. Longitudinal designs are recommended to confirm the direction of the relationships. Moreover, these are complex relationships that require further exploration through structural models. Lastly, we consider it relevant to include other variables in the study that have shown their importance in previous research.

## 5. Conclusions

Victimization, whether recognized or not, is related to distress. The lack of perception of bullying can lead to a lack of intervention and perpetuate the distress of the victims. Mental health professionals need to consider bullying as a factor of distress in adolescence. It is essential to expand protocols to include information on involvement in different bullying behaviors, both current and past. Additionally, it is necessary to refer victims to specialized resources that can address their mental health needs.

Therefore, clinical evaluation should consider previous bullying experiences and self-reported victimization behaviors. Moreover, clinical intervention must emphasize strengthening capacities that can help improve well-being, such as resilience, as a protective factor.

## Figures and Tables

**Table 1:** Distribution of the sample in the study groups, frequency (percentage).

	Gender		Age		
	Boys	Girls	Early Adolescence	Middle Adolescence	Late Adolescence
Non-Victims	304 (67.8)	373 (72.2)	182 (67.4)	426 (69.3)	69 (86.3)
Inconsistent Victims	6 (1.3)	10 (1.9)	3 (1.1)	12 (1.9)	1 (1.3)
Unperceived Victims	115 (26.0)	106 (20.3)	66 (24.5)	147 (23.9)	8 (10.1)
Consistent Victims	22 (4.9)	28 (5.6)	19 (7.0)	30 (4.9)	1 (1.3)

**Table 2:** Means (and SD) of distress according to perception of victimization and gender.

	Total	Boys	Girls
Non-Victims	1.84 (0.71)	1.65 (0.64)	1.99 (0.73)
Inconsistent Victims	1.99 (0.76)	1.65 (0.53)	2.19 (1.19)
Unperceived Victims	2.25 (1.01)	2.07 (0.75)	2.43 (0.72)
Consistent Victims	2.59 (0.93)	2.27 (0.99)	2.84 (0.81)

**Table 3:** Means (and SD) of distress according to perception of victimization and age.

	Early Adolescence	Middle Adolescence	Late Adolescence
Non-Victims	1.61 (0.55)	1.89 (0.73)	2.13 (0.82)
Inconsistent Victims	1.63 (0.75)	2.11 (1.09)	1.5
Unperceived Victims	2.09 (0.81)	2.29 (0.71)	2.67 (0.96)
Consistent Victims	2.29 (0.79)	2.77 (0.99)	2.90

**Table 4:** Correlations and means of study variables.

	1	2	3	4	5	6	7	8	9
Control variables									
1 Gender	-								
2 Age Victimization	−0.011	-							
3 Physical	−0.068 *	−0.099 **	-						
4 Verbal	−0.042	−0.071 *	0.427 ***	-					
5 Exclusion	0.066 *	−0.063 *	0.377 ***	0.546 ***	-				
6 Cyberbullying	−0.038	0.003	0.240 ***	0.415 ***	0.419 ***	-			
7 Previous	0.053	0.023	0.273 ***	0.384 ***	0.365 ***	0.282 ***	-		
8 Self nomination	0.013	−0.058	0.169 ***	0.354 ***	0.358 ***	0.255 ***	0.265 ***	-	
9 Resilience	−0.184 ***	−0.079 *	0.044	−0.149 ***	−0.086 **	−0.053	−0.239 ***	−0.118 ***	-
Dependent variable									-
10 Distress	0.210 ***	0.184 ***	0.127 ***	0.244 ***	0.197 ***	0.127 ***	0.368 ***	0.173 ***	0.334 ***
M boys			0.32	0.29	0.18	0.06			2.73
M girls			0.22	0.24	0.27	0.04			2.42
*t*			2.11 *	1.31	−2.06 *	1.18			5.66 ***
M Ea			0.31	0.32	0.29	0.04			2.68
M Ma			0.28	0.25	0.21	0.06			2.55
M La			0.10	0.17	0.17	0.05			2.42
*F*			2.65	2.35	1.83	0.58			2.24

Note: Gender: 1 = Boy; 2 = Girl; Self nomination: 0 = No perception; 1 = Victim perception. Ea—Early adolescence; Ma—Middle adolescence; La—Late adolescence. * *p* < 0.05; ** *p* < 0.01; *** *p* < 0.001.

**Table 5:** Linear regression of perception of victimization, bullying variables, and resilience on adolescent distress by gender.

Dimensions	Boys		Girls	
	*β*	IC 95%	*β*	IC 95%
Self report				
Physical victim	0.022	−0.075–0.116	0.037	−0.060–0.146
Verbal victim	0.148 **	0.026–0.264	0.037	−0.082–0.173
Exclusion victim	−0.106	−0.285–0.017	0.158 ***	0.123–0.558
Cyberbullying victim	0.065	−0.077–0.320	−0.066	−0.444–0.064
Previous victim	0.303 ***	0.645–1.258	0.253 ***	0.523–1.129
Self nomination	−0.018	−0.334–0.227	0.098 *	0.023–0.550
Resilience	−0.182 ***	−0.247–−0.081	−0.239 ***	−0.345–0.189
Nagelkerke R^2^	0.20		0.23	
*F*	14.41 ***		19.82 ***	

Note: * *p* < 0.05; ** *p* < 0.01; *** *p* < 0.001.

**Table 6:** Linear regression of perception of victimization, bullying variables, and resilience on adolescent distress by age.

Dimensions	Early Adolescence		Middle Adolescence		Late Adolescence	
	*β*	IC 95%	*β*	IC 95%	*β*	IC 95%
Self report						
Physical victim	0.025	−0.090–0.136	0.016	−0.073–0.107	−0.197	−0.829–153
Verbal victim	0.148	−0.006–0.271	0.038	−0.069–0.161	0.001	−0.717–0.717
Exclusion victim	0.033	−0.108–0.168	0.096 *	0.002–0.248	0.143	−0.244–0.621
Cyberbullying victim	0.038	−0.235–0.445	−0.069	−0.347–0.038	−0.180	−1.21–1.01
Previous victim	0.281 ***	0.482–1.337	0.278 ***	0.621–1.160	0.138	−0.377–1.31
Self nomination	0.002	−0.301–0.309	0.089 *	0.023–0.521	−0.146	−1.82–0.329
Resilience	−0.192 ***	−0.287–0.074	−0.209 ***	−0.285–0.131	−0.518 ***	−0.790–0.324
Nagelkerke R^2^	0.23		0.21		0.29	
*F*	11.38 ***		21.41 ***		5.21 ***	

Note: * *p* < 0.05; 0.01; *** *p* < 0.001.

## Data Availability

The datasets used and analyzed during the current study are available from the corresponding author on reasonable request.
